# Nutritional status of female prisoners in Antanimora prison, Madagascar

**DOI:** 10.11604/pamj.2019.33.119.18170

**Published:** 2019-06-17

**Authors:** Lantonirina Ravaoarisoa, Arivony Hanitriniaina Pharlin, Niaina Zakaria Rodolphe Andriamifidison, Roger Andrianasolo, Jean de Dieu Marie Rakotomanga, Julio Rakotonirina

**Affiliations:** 1 Institut National de Santé Publique et Communautaire, Faculté de Médecine d’Antananarivo, Antananarivo, Madagascar

**Keywords:** Female prisoner, nutritional status, diet, prison conditions

## Abstract

**Introduction:**

The prison population in low-income countries is a group vulnerable to undernutrition, particularly incarcerated women. The aim of the study is to assess the nutritional status of women in prison and to determine the social profile and prison conditions related to undernutrition.

**Methods:**

A cross-sectional study was conducted among 125 women prisoners in Antanimora prison located in the city of Antananarivo, Madagascar. All women detained for 3 months or more at the time of the survey were included in the study. Data collection was conducted in May and June 2013. A survey of women and anthropometric measurements were carried out to collect the data.

**Results:**

The proportion of undernourished female prisoners is 38.4%. Five percent of pregnant and lactating women and 44.3% of non-lactating and non-pregnant women are undernourished. The factors related to undernutrition of women prisoners are: taking two meals a day instead of three meals (p = 0.003), insufficient energy intake (p < 0.001), incarceration duration of more than 10 months (p < 0.001), absence of family visits (p = 0.013) and lack of financial assistance from family (p = 0.013).

**Conclusion:**

Improving the prisoners' diets and assistance from family both help to fight against prisoner undernutrition in prisons.

## Introduction

Prisoners are a population particularly vulnerable to undernutrition, even before incarceration. They are often in very precarious social situations, marginalized, without income and in a state of health weakened by chronic undernutrition. In addition to all of these stressors, their state of detention is often not in accordance with human rights [1]. In high-income countries, undernutrition in prisons is not a problem because the regulations concerning the diet of prisoners are generally respected [2]. In low-income countries, undernutrition is common in prisons. In 2005, more than half of the detainees in Mbanza Ngungu Prison in the Democratic Republic of Madagascar were seriously undernourished [3]. In Madagascar in 2010, 26% of male and female prisoners in eight prisons were affected by protein-energy malnutrition [4]. Women of reproductive age are a group vulnerable to undernutrition. According to the Demographic and Health Survey (DHS) conducted in 2008-2009 in Madagascar, 26.7% of women of reproductive age suffered from undernutrition (Body Mass Index, BMI less than 18.5 kg/m^2^) [5]. Incarceration can exacerbate this issue of undernutrition among women and thus increase its incidence and prevalence in prisons. This study was conducted to know the nutritional situation of women in prison in Madagascar. The objectives of the study are to assess the nutritional status of female prisoners and to identify the social profile and prison conditions related to undernutrition.

## Methods

A cross-sectional study was conducted among women prisoners in Antanimora prison. Antanimora prison is a penitentiary institution located in the city of Antananarivo, the capital of Madagascar. The prison accommodates all kinds of prisoners regardless of sex, age, or type of offense. The study includes all female prisoners of all ages who were detained for 3 months or more at the time of data collection. Data collection was conducted in May and June 2013. Information on social profile and prison conditions was collected by a survey of women using a pre-tested questionnaire. The 24-hour dietary recall method was used to calculate the energy intake of women's diets. Nutritional status was assessed by anthropometric measurements such as weight, height and Mid Upper Arm Circumference (MUAC). Women were weighed with a Seca digital scale (accurate within 100g). Height was measured with a Seca wall-mounted measuring rod (accurate within 1cm). MUAC was measured on the left arm, midway between the acromion and the olecranon with adult-specific measuring tape (accurate within 1mm). Nutritional status was estimated from BMI for non-pregnant women and from MUAC for pregnant women. BMI is calculated by dividing weight in kilograms by height in meters squared. Undernutrition is defined as a BMI less than 18.5 kg/m^2^ or a MUAC less than 220mm. The study was approved by the National Institute of Public and Community Health's Ethics Committee in Madagascar. The survey of women prisoners was carried out with the authorization of the Director of Prison Administration. Women's participation in the study was voluntary and their consent was requested after explaining the objectives and the course of the survey. The women were informed that participation in the investigation would have no impact on their prison life or on the judicial proceedings concerning their case. The investigation was anonymous and respected confidentiality and professional secrecy. The data was analyzed by Stata software. The usual descriptive analysis was completed. The Odds Ratio (OR) with a 95% Confidence Interval (CI) was calculated. The Chi-square test and Fischer's exact test were used to compare the proportions. The significance level is set at 0.05.

## Results

**Sample description:** a total of 125 female prisoners are included in the study. Table 1 shows the description of the social profile and the detention conditions of these women. The ages of the prisoners ranged from 16 to 62 years. The mean age (± SD) was 33.4 years (± 11.8) and three women were under 18 years old. At the time of the study, sixteen women (12.8%) were breastfeeding and three (2.3%) were pregnant.

**Table 1:** Social profile and detention conditions of female prisoners

Characteristic	n (%)
**Age (year)**	
≤ 24	37 (29.6)
25-39	48 (38.4)
40 et +	40 (32.0)
**Marital status**	
Married	71 (56.8)
Single	54 (43.2)
**Number of children**	
0	39 (31.2)
1-3	47 (37.6)
4 +	39 (31.2)
**Education level**	
Illiterate	15 (12.0)
Primary	47 (37.6)
Secondary	51 (40.8)
University	12 (9.6)
**Prior occupation**	
Primary sector	9 (7.2)
Secondary sector	37 (29.6)
Other*	79 (63.2)
**Détention status**	
Awaiting trial	73 (58.4)
Guilty verdict	52 (41.6)
**Duration of incarceration (months)**	
≤4	35 (28.0)
5-9	65 (52.0)
10 +	25 (20.0)
**Number of family visits (per month)**	
None	45 (36.0)
1-2	46 (36.8)
3 +	34 (27.2)
**Family financial assistance**	
Yes	56 (45.5)
No	99 (54.5)
**Number of meals per day**	
2	104 (83.2)
3	21 (16.8)
**Energy intake (kcal/day)**	
<1400	76 (60.8)
≥1400	49 (39.2)

* informal sector

**Nutritional status of women:** forty-eight women (38.4%) were undernourished (BMI < 18.5 kg/m^2^ or MUAC < 220mm). The 3 pregnant women included in the study were well-nourished and only 1 of the 16 breastfeeding women was undernourished. For the group of pregnant and lactating women, the prevalence of undernutrition is estimated at 5.3%. This prevalence was 44.3% for non-pregnant and non-lactating women, a significantly higher value (p = 0.001).

**Factors associated with the nutritional status:** Table 2 shows the association between women's social profile and their nutritional status. Among the studied characteristics, only the number of children was significantly associated with nutritional status. Women with more than three children were more frequently among undernourished compared to well-nourished (43.7% against 23.4%). The association between women's detention conditions and their nutritional status is displayed in Table 3. For undernourished women, the proportion of those with an incarceration duration more than 4 months, who had no family visit or financial assistance, with two meals a day and with a low total energy intake (< 1400 kcal per day) were significantly higher.

**Table 2:** Association of undernutrition and the social profile of female prisoners

Social profile	Undernutrition				OR [IC 95%]	p
	Yes (n=48)		No (n=77)			
	n	%	n	%		
**Age (year)**						
≤24	12	25.0	25	32.4	0.43 [0.15 – 0.21]	NS
25-39	15	31.2	33	42.9	0.41 [0.16 – 1.07]	
40 +	21	43.8	19	24.7	1	
**Marital status**						
Married	28	58.3	43	55.8	1.11 [0.50 – 2.45]	NS
Single	20	41.7	34	44.2	1	
**Number of children**						
0	14	29.2	25	32.5	1	0.042
1-3	13	27.1	34	44.1	0.68 [0.25 – 1.88]	
4 +	21	43.7	18	23.4	2.08 [0.76 – 5.73]	
**Education level**						
Illiterate & Primary	25	52.1	37	48.0	1	NS
Secondary	18	37.5	33	42.9	0.81 [0.35 – 1.86]	
University	5	10.4	7	9.1	NA^a^	
**Occupation before incarceration**						
Primary sector	2	4.2	7	9.1	NA^a^	NS
Secondary sector	13	27.1	24	31.2	0.76 [0.31 – 1.83]	
Other^b^	33	68.8	46	59.7	1	

a Not applicable

b Informal sector

**Table 3:** Association of nutritional status and women’s detention conditions

Detention condition	Undernutrition				OR [IC 95%]	p
	Yes (n=48)		No (n=77)			
	n	%	n	%		
**Detention status**						
Awaiting trial	27	56.2	46	59.7	1	NS
Guilty verdict	21	43.8	31	40.3	1.15 [0.52 – 2.55]	
**Duration incarceration (month)**						
≤4	5	10.4	30	39.0	1	<0.001
5 +	43	89.6	47	61.0	5.49 [1.81-17.80]	
**Number of family visits (per month)**						
0	25	52.1	20	26.0	3.00 [1.06 – 8.64]	0.013
1-2	13	27.1	33	42.8	0.95 [0.32 – 2.81]	
3 +	10	20.8	24	31.2	1	
**Family financial assistance**						
Yes	15	31.2	41	53.2	1	0.016
No	33	68.8	36	46.8	2.51 [1.10 – 5.74]	
**Number of meals per day**						
2	46	95.8	58	75.3	7.53 [1.56 – 9.44]	0.003
3	2	4.2	19	24.7	1	
**Energy intake (kcal/j)**						
≤1400	45	93.7	31	40.3	22.26 [5.89-8.83]	<0.001
>1400	3	6.3	46	59.7		

## Discussion

There were 280 women detained in Antanimora prison in 2013. The 125 women included in this study in May and June 2013 represent 45% of the women imprisoned for that year. The results obtained are assumed to reflect the situation of all female prisoners in the prison.

**Nutritional status of female prisoners:** the results of the study showed that 38.4% of women detained in Antanimora prison are undernourished (BMI < 18.5 kg/m^2^). According to the Demographic and Health Survey (DHS) carried out in 2008-2009 in Madagascar, the prevalence of undernutrition (BMI < 18.5 kg/m^2^) among women of reproductive age was 20.3% for the Analamanga region (the region in which the prison is located) and 26.7% at the national level [5]. This higher prevalence of undernutrition among incarcerated women in Antanimora prison compared to the female population as a whole confirms the vulnerability of prison populations to undernutrition. This public health issue is widespread in prisons in low-income countries [6]. Pregnant and lactating female prisoners seem to be less affected by undernutrition. For these categories, the proportion of women who were malnourished was 5.3% compared to 44.3% for non-lactating and non-pregnant women (p = 0.001). The existence of charitable associations and a nutrition program which provide food to pregnant and lactating women in Antanimora prison may partly explain this situation. It would be essential to know the nutritional status of women before incarceration to confirm that this high prevalence of undernutrition is related to incarceration. It was known that the prison selects vulnerable women even before incarceration [7]. Conducting a follow-up study of the nutritional status of women during incarceration could complement the results of this study.

**Factors associated with the nutritional status of female prisoners:** a number of incarceration conditions are associated with the nutritional status of female prisoners, including: duration of incarceration, prevalence of family visits and external financial assistance, number of meals per day and daily caloric intake from diet. All of these factors related to the nutritional status of female prisoners contribute to the single underlying variable that is diet. Diet is the main determinant of nutritional status [8]. Women who were able to consume three meals a day were more likely to be well-nourished (OR = 7.5 [1.6-9.4], p = 0.003), as well as those with a daily energy intake greater than 1400 kcal (OR = 22.3 [5.9-8.8], p < 0.001). In Antanimora prison, meals are only served once per day, consisting of one ladle of cassava per person. For 60% of prisoners, the food ration is not enough to provide the basal energy expenditure known as “basal metabolic rate” estimated at an average of 1400 kcal per day. This value is the minimum amount of vital energy that is essential for the functioning of the body's main vital organs (breathing, heartbeat, etc.) [9]. Insufficient caloric intake is common among prison populations in low-income countries. According to the United Nations Human Rights Committee, the energy allowance of prisoners in Madagascar is 800 kcal/day, 30% of the 2500 kcal necessary for maintaining basic health [10]. Across all of sub-Saharan Africa, the nutrition of female prisoners is suboptimal [11]. In comparison, prisons in high-income countries like France provide diets for prisoners that are well-balanced, of good quality and with adequate nutritional value [12].

Women who didn't receive family visits (OR = 3.0 [1.1-8.6], p = 0.013) and women who didn't benefit from financial assistance from their family (OR = 2.5 [1.1-5.7], p = 0.016) were significantly frequent among the undernourished female prisoners. In general, the family members of the women who visited them provided food or money that would allow the prisoners to improve the quality and quantity of their diets. An inadequate supply of food is a common problem in prisons in low-income countries. The state cannot sufficiently provide adequate amounts of high-quality food to the prisoners and therefore assistance from family members is important. However, it is often impossible for families to provide supplemental food for varying reasons. According to studies carried out by *Médecin Sans Frontière* in a prison in Guinea in 2009, two-thirds of the detainees received no visits from their families during their detention; some prisoners were even abandoned by their families and ate only the daily food provided by the prison [7]. Undernourished women have longer incarceration durations than well-nourished women. In fact, 90% of undernourished prisoners were incarcerated more than 5 months compared to 61% of well-nourished prisoners (p < 0.001). Remaining in prison for longer periods of time exposes the prisoners to unfavorable nutritional conditions which lead to a state of minor or severe undernutrition, depending on the resistance of the body. The reserve of nutrients that women have stored in their bodies before incarceration diminishes over time. The effect of incarceration duration in prison on the nutritional status of female prisoners is a result of exposure to inadequate diets over a long time period.

## Conclusion

The prevalence of undernutrition is high among women detained in Antanimora prison. Undernutrition is attributed to inadequate diet because the quantity and quality of food provided by the administration responsible for the prison was largely insufficient. Those who had the option to receive food from outside sources were less susceptible to undernutrition. Support by family and food aid programs are important ways to improve nutrition of prisoners.

### What is known about this topic

- The prison population in low-income countries is a group vulnerable to undernutrition because the regulations concerning the diet of prisoners are not generally respected;
- Women of reproductive age are a group vulnerable to undernutrition;
- Inadequate prison conditions are related to undernutrition.

### What this study adds

- Prevalence of undernutrition among female prisoners in Madagascar;
- Knowledge about diet of female prisoners in Madagascar;
- Identify factors related to undernutrition in prison.

## Competing interests

The authors declare no competing interests.
